# Aromatherapy Massage on the Abdomen for Alleviating Menstrual Pain in High School Girls: A Preliminary Controlled Clinical Study

**DOI:** 10.1155/2012/187163

**Published:** 2011-09-22

**Authors:** Myung-Haeng Hur, Myeong Soo Lee, Ka-Yeon Seong, Mi-Kyoung Lee

**Affiliations:** ^1^Department of Nursing, Eulji University, Daejeon 301-746, Republic of Korea; ^2^Brain Disease Research Center, Korea Institute of Oriental Medicine, Daejeon 305-811, Republic of Korea; ^3^Department of Nursing, Kimcheon Science College, Kimcheon 740-703, Republic of Korea; ^4^Department of Nursing, Sungshin Women's University, Seoul 142-100, Republic of Korea

## Abstract

This study investigated the alleviating effects of aromatherapy massage and acetaminophen on menstrual pain in Korean high school girls. Subjects were divided into two groups: the aromatherapy massage (treatment) group (*n* = 32) and the acetaminophen (control) group (*n* = 23). Aromatherapy massage was performed on subjects in the treatment group. The abdomen was massaged once using clary sage, marjoram, cinnamon, ginger, and geranium in a base of almond oil. The level of menstrual pain was assessed using a visual analogue scale at baseline and twenty-four hours afterward. The reduction of menstrual pain was significantly higher in the aromatherapy group than in the acetaminophen group. Using multiple regression, aromatherapy massage was found to be more highly associated with reduction in the level of menstrual pain than acetaminophen. These finding suggest that aromatherapy massage may be an effective treatment for menstrual pain in high school girls. However, it could not be verified whether the positive effects derived from the aromatherapy, the massage, or both. Further rigorous studies should be conducted using more objective measures.

## 1. Introduction

Menstrual pain is a common complaint, something that an estimated 25–97% of women experience [1]. Several complementary therapies have been used to treat menstrual pain, including acupuncture, herbal medicine, and spinal manipulation [2, 3]. Another option is aromatherapy massage [4].

Aromatherapy is the therapeutic use of essential oils derived from plants [5]. These oils can be absorbed into the body via the skin or the olfactory system [5]. Massage therapy can be defined as a mean of manipulating soft tissues using pressure and traction [6] and is reported as effective for menstrual pain [7–9]. Aromatherapy is generally performed as combining with massage, and it is thought to be safe and effective in treating menstrual pain or dysmenorrhea. Our previous study also showed that aromatherapy is effective in reducing the menstrual pain in college women [4]. This study was conducted to assess the comparative effects of aromatherapy massage and acetaminophen in menstrual pain relief of high school girls. 

## 2. Materials and Methods

Fifty-five high school girls with menstrual pain were recruited from the city of Daejeon in Korea through bulletin board and using flyer advertising to participate in aromatherapy massage session. Volunteers were eligible to participate in the study if their menstrual pain was measured at six or more points on a ten centimeter Visual Analogue Scale (VAS) and if they had no systemic disease or disease of the genital organs. 

The subjects were assigned to a treatment group to receive aromatherapy massage (*n* = 32) and a control group to receive acetaminophen (*n* = 23) according to their preferences. Ten participants dropped out of the aromatherapy massage group because of taking analgesics (8 with anxious about pain; 2 with no effect of aromatherapy massage). No one dropped out of the control group. The experimental protocol was approved by the Review Board of the University hospital. The outcome measures were the pain level as measured with a VAS before intervention and twenty-four hours afterwards. Subjects in the treatment group received one ten-minute abdominal massage using essential oils: clary sage, marjoram, cinnamon, ginger, and geranium in a 1 : 1 : 0.5 : 1.5 : 1.5 ratio, diluted in almond oil with a final concentration of 5%. Subjects in the control group received no additional treatment. 

Data were analyzed using SigmaStat (Systat Software, CA) and SPSS (SPSS Inc, Chicago, IL). Since the data were not normally distributed, the results are presented as medians and interquartile ranges (IQRs). All outcomes were compared using the nonparametric Mann-Whitney U test between groups. Multiple regression analyses were used to estimate the effects and the validity of the hypothesis. The change score of pain (twenty-four hours after intervention minus baseline) was regressed on the baseline score. 

## 3. Results

The groups did not differ significantly in age, age of menarche, duration, and amounts of menstruation (data were not shown here). 

**Figure 1:**
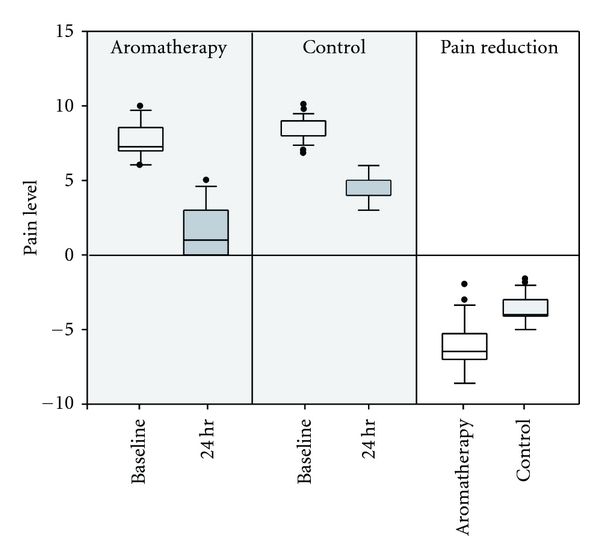
Pain level: baseline and twenty-four hours after intervention, and pain reduction (twenty-four hours minus baseline) due to aromatherapy massage and acetaminophen. The results are presented as medians and interquartile ranges (IQRs). Outliers are indicated by ●.

Figure 1 shows the medians and IRQs for the level of menstrual pain in both groups measured at baseline and twenty-four hours afterwards. The pain was significantly reduced in the aromatherapy massage treatment group over the control group. Because of baseline differences, we regressed the changes of pain level against the baseline pain score. Aromatherapy massage was strongly associated with reduction of pain (beta = −3.07, 95% confidence interval −3.83 to −2.29, *t* = −8.00, *P* < 0.001), followed by the baseline value of level of pain (beta = −0.69, 95% confidence interval −1.07 to −0.31, *t* = −3.69, *P* < 0.001). This difference favored the treatment group.

## 4. Discussion

This preliminary controlled clinical trial was conducted to investigate the efficacy of aromatherapy massage on menstrual pain compared to acetaminophen. Subjects in the aromatherapy massage group showed greater reduction of pain after twenty-four hours compared with subjects in the control group who took acetaminophen. This result supports previous findings that aromatherapy massage alleviates menstrual pain compared with placebo control [4]. 

Oral analgesics, such as acetaminophen, are widely used for managing pain. However, frequent dosing is required, and drug interaction or other adverse effects such as liver toxicity may occur. The availability of abdominal massage with essential oils, which has no adverse effects, makes this therapy attractive, especially if it can be shown to be as efficacious as other options.

Menstrual pain is caused by reduced blood flow due to uterine hyperactivity [1]. It can occur when the menstrual flow is constricted and can be relieved by increased blood circulation and antispasmodics. Topically applied diluted essential oils in combination with abdominal massage may be beneficial in improving blood circulation [5]. The scent of the essential oils activates the olfactory senses, which triggers the limbic system. This may be effective in helping to alleviate menstrual pain. 

There are several limitations to this study, one of which is high dropout rate from the aromatherapy massage group due to anxiety about pain (8 subjects) and noneffectiveness (2 subjects). Another concern is the nonrandomized methods, which may result in selection bias and false positive findings. A further limitation is that only one trial was conducted to test the efficacy of aromatherapy massage. Moreover, this study lacked one more equivalent treatment control group to estimate the superior effectiveness of aromatherapy massage. Therefore, it is not clear whether the positive effects were due to the aromatherapy, the massage, or both (e.g., identical results may have been achieved by using only massage or by using aromatherapy and massage). 

In conclusion, our data suggests that aromatherapy may be effective in alleviating menstrual pain. However, we cannot completely elucidate the nonspecific effects of aromatherapy due to the lack of placebo control. Further randomized studies should be carried out with appropriate placebo controls. These should include more objective measures, such as hormonal changes associated with menstruation to explain the possible mechanism of reduction in menstrual pain.
